# Use of heart failure medications in older individuals and associations with cognitive impairment

**DOI:** 10.1186/s12877-023-04223-3

**Published:** 2023-08-29

**Authors:** Linnea Abramsson, Annica Backman, Hugo Lövheim, David Edvardsson, Maria Gustafsson

**Affiliations:** 1 Department of Integrative Medical Biology, Umeå University, Umeå, 901 87 Sweden; 2 Department of Nursing, Umeå University, Umeå, 901 87 Sweden; 3 Department of Community Medicine and Rehabilitation, Geriatric Medicine, Umeå University, Umeå, Sweden; 4 Wallenberg Center for Molecular Medicine, Umeå, Sweden; 5 School of Nursing and Midwifery, La Trobe University, Bundoora, Vic, 3084 Australia

**Keywords:** Cognitive impairment, Heart failure, Target doses, Filled prescriptions

## Abstract

**Background:**

To achieve the best treatment of heart failure, it is important to use all recommended drugs at their target doses. Given that underuse of medications can occur in individuals with cognitive impairment, we investigated the filled prescriptions and target doses of heart failure medication for older individuals with and without cognitive impairment as well as associated factors.

**Methods:**

The study was based on two separate datasets. The first dataset, which was based on data from questionnaires sent to nursing homes in Sweden, included 405 individuals with heart failure. The data were linked with the Swedish Prescribed Drug Register and the National Patient Register to obtain information regarding filled prescriptions of heart failure medications and heart failure diagnoses among the population. In the second dataset, medical records of individuals aged 75 years or older admitted to a hospital in northern Sweden were reviewed and individuals with heart failure were identified. Target doses of heart failure medications were evaluated in 66 individuals who lived at home.

**Results:**

Filled prescriptions of mineralocorticoid receptor antagonists and loop diuretics were significantly more common in individuals without cognitive impairment (OR 1.087; 95% CI 1.026–1.152, p < 0.05) and (OR 1.057; 95% CI 1.017–1.098, p < 0.05), respectively. There were no significant differences between individuals with and without cognitive impairment in terms of achieving target doses for any of the drug classes. A higher age was associated with fewer filled prescriptions and less ability to reach the target doses of beta blockers (OR 0.950; 95% CI 0.918–0.984, p < 0.05) and (OR 0.781; 95% CI 0.645–0.946, p < 0.05), respectively.

**Conclusions:**

Our results suggest that individuals with cognitive impairment are partly undertreated for heart failure in that they had fewer filled prescriptions of important heart medications. Separately, the relatively low proportion of older individuals reaching target doses is an important observation and indicates that treatment of heart failure could be further optimised among older individuals.

**Supplementary Information:**

The online version contains supplementary material available at 10.1186/s12877-023-04223-3.

## Background

Heart failure is a disease that leads to high mortality and a decreased quality of life. Depending on the definition of heart failure, its prevalence is ~ 10% among people 70 years or older in developed countries. However, significant advances have been made in its treatment in recent years and the pharmacological guidelines have been revised [1, 2]. Pharmacological treatment with an angiotensin converting enzyme inhibitor (ACEI), a beta blocker (BB), and a mineralocorticoid receptor antagonist (MRA) have a class I recommendation for all individuals with heart failure with reduced ejection fraction (HFrEF) or with mildly reduced ejection fraction (HFmrEF), with New York Heart Association (NYHA) II–IV [1]. In 2021, sodium-glucose transport protein 2 (SGLT2)-inhibitors also received a class I recommendation for individuals with HFrEF, NYHA II–IV. For individuals with persistent symptoms, ACEI is recommended to be replaced with an angiotensin receptor-neprilysin inhibitor (ARNI). For individuals who do not tolerate ACEI or ARNI, an angiotensin receptor blocker (ARB) is recommended.

Both ACEI and BB reduce mortality and hospitalisation caused by heart failure and reduce the symptoms compared with a placebo in individuals with heart failure [3–6]. ACEI has also shown positive effects on quality of life in this population [4]. The addition of MRA further reduces hospitalisation due to heart failure and decreases mortality [7]. SGLT2 inhibitors can further reduce cardiovascular mortality [8]. ARNI has recently been recommended to replace ACEI before the use of an ARB because ARNI can reduce total mortality in contrast to ARB [1, 9, 10]. ARNI, like ACEI, also has positive effects on quality of life [9]. Besides these drugs with a class I recommendation, loop diuretics, ivabradine, or digoxin can be added for individuals with persistent symptoms of heart failure [1]. Diuretics improve exercise tolerance and decrease hospitalisations due to heart failure [11], ivabradine decreases the combined measures of cardiovascular mortality and hospitalisations due to heart failure [12], and digoxin reduces the risk for hospitalisation due to heart failure [13].

The use of two or more drug classes with class I recommendation is associated with an additive effect in reducing mortality and decreasing rates of rehospitalisation in individuals with heart failure, according to a meta-analysis [14]. In addition to the importance of the number of heart failure medications prescribed, reaching the target doses of heart failure medications is recommended to achieve the maximum effect of the drugs. Previous studies have found that many patients with heart failure do not reach the target doses of their prescribed drugs [15, 16]. Target doses of ACEI/ARB and BB were prescribed for 25–69% of individuals with heart failure according to one study [15], while another study found that only 33–35% of individuals with heart failure with BB prescriptions achieved their target dose [16]. However, the population with HFrEF has become larger and older and is at higher risk for adverse events. Many of the studies performed regarding heart failure treatment have also excluded multimorbidity and frail individuals, thereby limiting the evidence for this population [17].

The risk for cognitive impairment increases with age [18]. In addition, there is a comorbidity between heart failure and cognitive impairment. According to a meta-analysis of 4,176 individuals with heart failure, 43% had cognitive impairment as a comorbidity [19]. Previous studies have found that individuals with cognitive impairment are undertreated for some cardiovascular diseases [20, 21] and that individuals with concomitant heart failure and cognitive impairment have a high risk of drug-related hospital admissions [22].

To achieve the optimal treatment of heart failure, it is important to use all the recommended drugs and to reach the target doses of these drugs. Given that undertreatment occurs in individuals with cognitive impairment, we evaluate whether there are differences in the prescription of drugs for heart failure between those with and without cognitive impairment.

## Aim

The first aim of this study was to investigate the filled prescriptions of each drug class of heart failure medication in individuals with and without cognitive impairment. The second aim was to evaluate any possible differences between individuals with and without cognitive impairment in terms of whether they reached the target doses of their heart failure medications. A third aim was to investigate factors associated with filled prescriptions and target doses.

## Methods

This study was based on two separate datasets. The first dataset (dataset 1) was based on a survey and included older individuals living in nursing homes. The second dataset (dataset 2) was based on a randomised controlled study, and the population included older individuals living at home. The different populations are described below.

### Data based on a survey

Dataset 1 was based on a survey from the Swedish National Inventory of Care and Health in Residential Aged Care (SWENIS), which was sent randomly to a nationally representative sample of nursing homes in Sweden in 2018–2019. A total of 315 nursing homes were contacted for participation, and 235 agreed to participate. In total, 7073 questionnaires were sent to these 235 nursing homes, and the final sample included 3894 residents (response rate of 55%) from 187 nursing homes. The surveys were completed by staff at the nursing home who had the best knowledge of individuals. The survey included, for example, questions regarding demographic information, quality of life according to EuroQuol five dimension (EQ-5D) [23], assessments of functioning in the activities of daily living (ADL) [24], and cognition. Cognitive function was classified according to Gottfries’ cognitive scale with 27 items, where a score < 24 is classified as cognitive impairment [25]. This correlates with 90% sensitivity and 91% specificity [24] to the cut-off of 24/30 used in the Mini-Mental State Examination [26].

In the present study, the data from the survey were linked with the Swedish Prescribed Drug Register, the Swedish Cause of Death Registry, and the National Patient Register through personal identity numbers to obtain information regarding heart failure medications and heart failure diagnoses among the population.

#### Study population

Of the 3894 individuals who completed and returned the questionnaires, 2771 individuals had complete personal identity numbers and could be linked to the Swedish Prescribed Drug Register. Data regarding filled prescriptions for heart failure medication were collected for the period January 01–June 30, 2019. The Swedish Cause of Death Register was used to select individuals alive on June 30, 2019. Of the 2479 living individuals, those with heart failure according to International Classification of Diseases ICD-10 code I50 [27] were selected. A total of 411 individuals had a heart failure diagnosis, but 6 individuals who did not have their cognitive function graded were excluded from the study, and thus 405 individuals were included in the analysis.

#### Definitions

Drug use was defined as one or more filled prescriptions during a 6-month timeframe. Drugs included in the analysis were agents acting on the renin-angiotensin system (Anatomical Therapeutic Chemical (ATC) classification C09), recommended BBs at heart failure (bisoprolol, carvedilol, and metoprolol) [1], MRA (C03DA), loop diuretics (C03C), digoxin (C01AA05), and ivabradine (C01EB17). SGLT2-inhibitors were not included in the analysis because the recommendation was not yet introduced during the inclusion period for dataset 1 and for most of the time of dataset 2.

### Data based on an ongoing randomised controlled study

Dataset 2 was based on an ongoing randomised controlled study of 700 individuals in the University Hospital in Umeå [28]. The study included individuals ≥ 75 years old living at home, listed at predefined health care units, and hospitalised at an emergency medical ward or an orthopaedic ward. During the first interview at the ward, each individual was classified by cognitive function according to a shortened four-item version of Gottfries’ cognitive scale [28, 29]. A score < 3 was classified as cognitive impairment. Medication adherence according to the Medication Adherence Report Scale (MARS-5) [30] and quality of life according to the EQ-5D were also assessed during the first interview. The individuals were randomised to the intervention group or control group when discharged from the hospital [28]. The intervention group received an extensive clinical pharmacy service for 180 days including *medication reconciliation* every second week and medication phone interviews at 7, 30, and 60 days after discharge from the hospital. The pharmacists discussed drug related problems with the primary care physician. The control group received standard care, including medication reviews at the ward from a clinical pharmacist. The primary aim was to investigate whether the intervention during the transition from hospital care to primary care could reduce the risk of drug-related hospital readmission within 180 days after discharge from the hospital. The inclusion of the individuals started in September 2018 and is still ongoing.

#### Study population

In the present study, the first 271 individuals from both the intervention and control group were included (2018-09-20 through 2021-09-14). Of these 271 individuals, those diagnosed with heart failure and at least one of following prescribed drugs were analysed: agents acting on the renin-angiotensin system (ATC classification C09), BBs recommended at heart failure (bisoprolol, carvedilol, and metoprolol), and MRA (C03DA). To exclude people with heart failure with preserved ejection fraction, (HFpEF) one inclusion criteria was to have at least one heart failure drug prescribed. Heart failure diagnosis was identified by ICD-I50 or notes of heart failure diagnosis in the medical record. Only individuals who already had a heart failure diagnosis on the day of admission were included in the study because the analysed medication lists were the individuals’ current medications at the time of admission to the hospital. Of the 271 individuals, 70 had a heart failure diagnosis at admission, and 68 of these individuals had at least one of the above-mentioned drugs prescribed. The exclusion criterion was individuals with a diagnosis of right ventricular failure with preserved left ventricular function. Two of the 68 individuals were excluded according to this criterion. Thus, 66 individuals were included in the current analysis.

#### Definitions

Information about the population was obtained from the medical records and included the individuals’ medication list when included in the previous study, laboratory values, admission notes, doctors notes, and discharge summary. Any readmission note was not analysed. The medication lists at inclusion to the study, i.e., at admission to hospital, were analysed according to whether the individual had reached the target doses of the prescribed drugs. Target doses for the medications were analysed according to European Society of Cardiology (ESC) guidelines [1, 2, 31]. For MRA, target doses according to Västerbotten County Council’s guidelines were used. Target doses were analysed for drugs with ATC classification C09 (captopril, enalapril, lisinopril, ramipril, candesartan, losartan, and sacubitril/valsartan), C07 (bisoprolol, carvedilol, and metoprolol), and C03DA (eplerenone and spironolactone). Target doses of the analysed drugs are presented in Supplementary Table S1.

For individuals who did not reach target doses, laboratory values and notes from the medical record were analysed to identify any possible reason for not reaching the target dose. The values analysed were renal function, potassium level, heart rate, blood pressure, and other apparent contraindications for the drugs. In cases where possible information regarding contraindications was missing from the notes, the following thresholds were used: a relative estimated glomerular filtration rate (eGFR) < 30 mL/min/1.73 m^2^ or a serum potassium level of > 5.5 mmol/L were classified as a possible reason for not reaching target dose of ACEI/ARB/ARNI and MRA [32], a heart rate < 60 was classified as a possible reason for not reaching target doses of BB [33], and a systolic blood pressure of < 90 mmHg [34] or an orthostatic blood pressure (defined as ≥20 fall in systolic pressure or ≥10 mmHg in diastolic pressure with standing) [35] was classified as a possible reason for not reaching the target dose of any of the drugs.

### Statistics

To analyse if there was a difference between individuals with and without cognitive impairment and drug use, a chi-squared test was used for both datasets. To adjust for other factors associated with drug use, multiple logistic regression models were constructed in both datasets. In dataset 1, filled prescriptions of each drug class was the dependent variable. The independent variables were cognitive function (Gottfries’ cognitive scale as a continuous variable), sex, age, EQ-5D, and ADL dependency. In the multiple regression model in dataset 2, the achieved target dose of each drug class was the dependent variable. The independent variables were cognitive function (the short version of Gottfries’ cognitive scale as a dichotomous variable), sex, age, and EQ-5D. For ACEI/ARB/ARNI and MRA, the potassium level and relative eGFR were also independent variables in dataset 2. Relative eGFR was estimated using the Chronic Kidney Disease Epidemiology Collaboration equation [36]. The statistical program used was Statistical Package for the Social Sciences for Windows, Version 25. We considered p-values < 0.05 significant for all statistical tests, and odds ratios were calculated with 95% confidence intervals.

## Results

The basic characteristics of the populations are presented in Table 1. In dataset 1, 405 individuals were included. More than half of the individuals were women, and the mean age was 86.8 years. Of the included individuals, 55% had cognitive impairment according to Gottfries’ cognitive scale, and the mean score was 20.0. In dataset 2, 66 cases were included in the analysis with a mean age of 86.0 years. Of these, 37 were women and 30% had a cognitive impairment classification according to the short version of Gottfries’ cognitive scale.

**Table 1:** Basic characteristics of the populations

	Dataset 1	Dataset 2
Cases *n*	405	66
Women *n (%)*	253 (62.5)	37 (56.1)
Mean age ± SD	86.8 ± 7.5	86.0 ± 6.14
Cognitive impairment *n (%)*	223 (55.1)^a^	20 (30.3)^b^
EQ-5D score (0–100), mean ± SD	64.5 ± 21.4	47.9 ± 21.7
Relative eGFR (mL/min/1.73 m^2^)		47.8 (21.3)
ADL score (0–6), mean ± SD	3.4 ± 2.1	
Gottfries’ score (0–27), mean ± SD	20.0 ± 7.4	

SD (standard deviation), ADL (activities of daily living), EQ-5D (EuroQuol five dimension). ^a^According to Gottfries’ original scale. ^b^According to Gottfries’ short version

BB and loop diuretics were the most common drug classes in dataset 1 in both individuals with and without cognitive impairment (Table 2). BB, MRA, and loop diuretics were all significantly more common in individuals without cognitive impairment. For agents acting on the renin-angiotensin system and digoxin, no significant differences were observed between the groups. None of the individuals had ivabradine as a filled prescription. When adjusting for different factors, the association with cognitive impairment was significant only for MRA and loop diuretics. There was also a significant association between lower age and filled prescriptions of BB. All results from the multiple logistic regression analysis from dataset 1 are shown in Table 3.

**Table 2:** Prevalence of heart failure medication in individuals with and without cognitive impairment (Dataset 1)

Drug	Individuals with cognitive impairment, *n (%)* *n = 223*	Individuals without cognitive impairment, *n (%)* *n = 182*	p-value
ACEI/ARB/ARNI	110 (49.3)	93 (51.1)	0.723
BB^a^	128 (57.4)	129 (70.9)	0.005
MRA	24 (10.8)	35 (19.2)	0.016
Loop diuretics	139 (62.3)	138 (75.8)	0.004
Digoxin	20 (9.0)	14 (7.7)	0.645
Ivabradine	0 (0)	0 (0)	-

ACEI (angiotensin converting enzyme inhibitor), ARB (angiotensin II receptor blocker), ANRI (angiotensin receptor-neprilysin inhibitor), BB (beta blocker), MRA (mineral corticoid receptor antagonist). ^a^ Bisoprolol, carvedilol, metoprolol

**Table 3:** Multiple logistic regression analysis of factors associated with different drug classes (Dataset 1)

	Odds ratio	95% confidence interval	p-value
***ACEI/ARB/ARNI***			
Female sex	1.205	0.756–1.921	0.434
Higher age	1.001	0.971–1.032	0.950
ADL score	1.011	0.894–1.142	0.865
Cognitive score^a^	1.009	0.974–1.045	0.633
EQ-5D	0.995	0.984–1.005	0.317
***BB*** ^b^			
Female sex	1.241	0.756–2.037	0.393
Higher age	0.950	0.918–0.984	0.004
ADL score	0.966	0.848-1.100	0.601
Cognitive score^a^	1.031	0.993–1.070	0.108
EQ-5D	1.003	0.992–1.014	0.620
***MRA***			
Female sex	1.362	0.707–2.625	0.355
Higher age	0.990	0.951–1.031	0.644
ADL score	1.070	0.901–1.269	0.441
Cognitive score^a^	1.087	1.026–1.152	0.005
EQ-5D	0.994	0.980–1.008	0.399
***Loop diuretics***			
Female sex	0.855	0.509–1.436	0.554
Higher age	1.020	0.986–1.055	0.255
ADL score	0.993	0.868–1.137	0.923
Cognitive score^a^	1.057	1.017–1.098	0.005
EQ-5D	0.997	0.985–1.008	0.559
***Digoxin***			
Female sex	1.501	0.640–3.520	0.350
Higher age	1.002	0.950–1.057	0.936
ADL score	0.912	0.735–1.131	0.401
Cognitive score^a^	1.021	0.960–1.086	0.511
EQ-5D	0.990	0.973–1.007	0.228

ACEI (angiotensin converting enzyme inhibitor), ARB (angiotensin II receptor blocker), ANRI (angiotensin receptor-neprilysin inhibitor), ADL (activities of daily living), BB (beta blocker), EQ-5D (EuroQuol five dimension), MRA (mineral corticoid receptor antagonist). ^a^ Gottfries’ cognitive scale, original version as a continuous variable. ^**b**^Bisoprolol, carvedilol, metoprolol

Of the population in dataset 2, 91% were prescribed a BB, 80% percent were prescribed an ACEI/ARB/ARNI, and 41% were prescribed an MRA (Table 4). Of all individuals included in dataset 2, 79% had at least two of the drug classes prescribed, and one-third had all three drug classes prescribed. In Table 4, the proportions of individuals reaching target doses are also presented. In total, 12 individuals reached target doses of ACEI/ARB/ARNI, 9 individuals reached target doses of BB, and 18 individuals reached target doses of MRA. In terms of percentages, most individuals were able to reach target doses of eplerenone, ramipril, and spironolactone. None of the individuals reached target doses of losartan, sacubitril/valsartan, or carvedilol. There were no significant differences in reaching target doses for any of the drug classes between individuals with and without cognitive impairment according to the chi-squared test.

**Table 4:** Individuals reaching target dose of each drug and relation to cognitive impairment (Dataset 2)

Drug	n (%) with each drug	Individuals reaching target doses, *n (%)*	Individuals with cognitive impairment, *n (%)* *n = 20*	Individuals without cognitive impairment, *n (%)* *n = 46*	p-value
*ACEI/ARB/ARNI n (%)*	53 (80.3)	12 (22.6)	4 (20.0)	8 (17.4)	0.064
Enalapril	13	4 (30.8)			
Ramipril	3	2 (66.7)			
Candesartan	24	6 (9.1)			
Losartan	12	0 (0)			
Sacubitril/valsartan	1	0 (0)			
*BB* ^*a*^ *n (%)*	60 (90.9)	9 (15.0)	1 (5.0)	8 (17.4)	0.178
Bisoprolol	27	3 (11.1)			
Carvedilol	1	0 (0)			
Metoprolol	32	6 (18.8)			
*MRA n (%)*	*27 (40.9)*	*18 (66.7)*	6 (30.0)	12 (26.1)	0.108
Eplerenone	9	7 (77.8)			
Spironolactone	18	11 (61.1)			

ACEI (angiotensin converting enzyme inhibitor), ARB (angiotensin II receptor blocker), ANRI (angiotensin receptor-neprilysin inhibitor), BB (beta blocker), MRA (mineral corticoid receptor antagonist). ^a^ Bisoprolol, carvedilol, metoprolol

The multiple logistic regression analysis in dataset 2 showed that the only significant association was between younger age and reaching the target dose of BB (Table 5).

**Table 5:** Multiple logistic regression analysis of factors associated with target doses of different drug classes (Dataset 2)

	Odds ratio	95% confidence interval	p-value
*ACEI/ARB/ARNI*			
Female sex	2.302	0.526–10.067	0.268
Higher age	0.988	0.860–1.135	0.868
Cognitive impairment^a^	1.194	0.240–5.941	0.828
EQ-5D	0.978	0.944–1.013	0.214
Potassium level	2.312	0.696–7.682	0.171
eGFR	1.026	0.987–1.066	0.199
*BB* ^b^			
Female sex	0.728	0.129–4.120	0.720
Higher age	0.781	0.645–0.946	0.011
Cognitive impairment^a^	1.863	0.166–20.881	0.614
EQ-5D	1.024	0.982–1.068	0.272
*MRA*			
Female sex	1.075	0.285–4.063	0.915
Higher age	0.971	0.861–1.096	0.636
Cognitive impairment^a^	1.089	0.246–4.810	0.911
EQ-5D	0.983	0.951–1.016	0.316
Potassium level	3.220	0.822–12.613	0.093
eGFR	1.000	0.965–1.035	0.989

ACEI (angiotensin converting enzyme inhibitor), ARB (angiotensin II receptor blocker), ANRI (angiotensin receptor-neprilysin inhibitor), BB (beta blocker), eGFR (estimated glomerular filtration rate), EQ-5D (EuroQuol five dimension), MRA (mineral corticoid receptor antagonist). ^a^Gottfries’ cognitive scale, short version as a dichotomous variable. ^b^Bisoprolol, carvedilol, metoprolol

Of the 41 individuals who did not reach target doses of ACEI/ARB/ARNI, possible reasons were identified in 12 cases. In 8 cases, the renal function was below 30 mL/min/1.73 m^2^), and therefore a possible reason for not reaching target doses was due to decreased renal function. One of these eight individuals had decreased renal function in combination with a high serum potassium level. Four of the individuals where a possible reason was found had hypotension or orthostatic hypotension. Of the 29 remaining individuals, no possible reason was identified.

In those with BB prescriptions, 51 individuals did not reach target doses. Possible reasons were found in seven of these individuals. Of these seven, five had hypotension or orthostatic hypotension and two had bradycardia. No possible reasons why target doses were not achieved were identified in the 44 remaining individuals.

Of the nine individuals who did not achieve the target dose of MRA, a reason was identified for only one individual, which was orthostatic hypotension.

## Discussion

In this study we found that individuals with cognitive impairment were less likely to receive MRA and loop diuretics. Further, there were no significant differences in reaching target doses for any of the drug classes between individuals with and without cognitive impairment. A higher age was associated with fewer filled prescriptions and less ability to reach target doses of BBs.

Undertreatment of individuals with cognitive impairment was observed in previous research [20, 21], but in the present study undertreatment was not observed with ACEI/ARB/ARNI, BB, or digoxin. The prescription of ACEI/ARB/ARNI and BB to individuals with cognitive impairment might have increased during recent years. A previous study found that cognitive impairment was associated with under-prescription of ACE/ARB and BB in 2007, but between 2007 and 2013 an improvement in treatment with these drugs in individuals with cognitive impairment was observed [37]. However, no significant difference in MRA prescription was observed in this population during this period. One possible reason for the difference between these drug groups could be that physicians are somewhat more restrictive in prescribing according to the latest recommendations for individuals with cognitive impairment. Agents that affect the renin-angiotensin system and BB have been recommended as class I recommendations for heart failure for several years [38, 39], but immediate prescription of both MRA, SGLT2 inhibitors, agents that affect the renin-angiotensin system, and BB was not implemented in ESC guidelines until 2021 [1, 2]. In the guidelines from 2016, prescription of MRA was only recommended if the individual still had symptoms or still had an ejection fraction ≤ 35% despite treatment with agents that affect the renin-angiotensin system and BB [2]. The other group of drugs that individuals without cognitive impairment more often received in this study was loop diuretics. This is somewhat surprising because loop diuretics might be prescribed more for individuals with insufficient treatment of heart failure to improve their symptoms. One possible explanation for this might be that individuals without cognitive impairment might be more likely to report symptoms and request symptomatic treatment.

In the present study, no significant differences in reaching target doses were seen between individuals with and without cognitive impairment. The study population may be too small, and even if there was a true difference between individuals with and without cognitive impairment, the number of individuals may be too limited to detect it. What we found in this study, however, was that a relatively low proportion of the individuals reached target doses of heart failure medications. In the relatively few cases where a reason for not achieving a target dose was identified, the most common was decreased renal function for ACEI/ARB/ARNI and hypotension/orthostatic hypotension for BB and MRA. Another possible reason for not reaching target doses of heart failure medications is treatment with other blood pressure lowering agents, such as calcium channel blockers [1], which lower the blood pressure and give less ability to increase the dose of heart failure medication. Additionally, a clinician’s definition of hypotension as a contraindication to reaching target dosing is likely higher than the definition used in this study (< 90 mmHg). In the present study, other blood pressure-lowering agents were not included in the analyses. Target doses of MRA and of ACEI/ARB/ARNI were achieved in 67% and 23% of the individuals, respectively, but regarding BB, target doses were only achieved in 15% of the individuals, which was lower than what was determined in a previous study [16]. The regression analyses in the present study showed that older individuals were not as likely to have a filled prescription of BB or to reach target doses of BB. This is consistent with earlier research [37, 40]. However, a previous study found that BBs decrease mortality in older individuals regardless of dose, but more research is needed to confirm this [41]. A probable reason for under-prescribing BBs to older individuals are the adverse effects, such as bradycardia and orthostatic hypotension, associated with these drugs [42, 43]. The prescribing physicians might therefore be more cautious about prescribing BB and increasing doses for older individuals. In addition, older individuals are generally underrepresented in clinical trials of heart failure medications, and the reduced risk in mortality is not as established as in younger individuals [17]. However, a benefit of receiving a BB after admission in terms of reduced rehospitalisations and mortality in individuals ≥ 75 years with heart failure was observed in one study [17]. Considering the adverse effects associated with these drugs, it is important to carefully follow-up the individual’s response to treatment. Nevertheless, the results show that the treatment of heart failure could be further optimised among older individuals.

No other factor than age was associated with reaching target doses in the regression analysis. Surprisingly, there was no found association between target doses of ACEI/ARB/ARNI and a lower eGFR. The use of these drugs in individuals with severely reduced renal function may cause undesirable outcomes such as hyperkalemia [44]. Although decreased renal function was identified as one reason for not reaching target doses, the study population may be too small to detect an association.

A major limitation to this study is that detailed heart failure diagnoses were missing in both datasets. We do not know if the diagnoses were based on echocardiography, and especially older people might receive the diagnosis of heart failure without objective findings. In dataset 1, all individuals with heart failure according to ICD-10 code I50 were included [27]. It would be more desirable to include only individuals with NYHA II-IV and HFrEF or HFmrEF because these are the individuals with indications for heart failure medications [1]. The broader group in dataset 1 has implications for the findings in this study. Further, we had no data regarding frailty, which might have affected the prescription of drugs. Also, if a major neurocognitive disorder (NCD) diagnosis had been used instead of Gottfries’ cognitive scale to identify people with and without cognitive impairment, the results may have been different. Gottfries’ cognitive scale was used in the present study to also include those people who suffer from cognitive impairment but have not yet received a major NCD diagnosis.

For MRA, a lower target dose was recommended in Region Västerbotten’s guidelines compared with ESC [1, 31]. For these drugs, Region Västerbotten’s target doses were used because most of the individuals had the drugs prescribed in this region. SGLT2 inhibitors were introduced to all individuals with heart failure NYHA II-IV and HFrEF or HFmrEF in the ESC guidelines for heart failure in 2021 [1, 2]. Because the recommendation was not yet introduced during the inclusion period in dataset 1 or for most of the time in dataset 2, this drug class was not included in the analysis.

In dataset 2, relative eGFR is used to find possible reasons for why individuals did not reach target doses. For drug dosing, absolute eGFR is recommended because it is a more accurate estimation of an individual’s renal function [45]. To estimate absolute eGFR, information regarding weight and height is necessary, and in most cases this information was missing. For ACEI/ARB/ARNI, decreased renal function is not an absolute contraindication for target doses of these drugs. In our analysis, a limit of eGFR < 30 mL/min/1.73 m^2^ was used as a possible reason for not reaching target doses. This, in combination with relative eGFR instead of absolute eGFR, is a limitation in explaining why individuals did not reach target doses. Another limitation in the present study is that the values of eGFR, potassium levels, heart rate, and blood pressure were non-recurring values. Further, a systolic blood pressure of < 90 mmHg used as a definition in this study is not adapted for an elderly person with cognitive impairment. In analysing the possible reasons for not reaching target doses, the number of individuals was too small to evaluate possible differences between individuals with and without cognitive impairment.

## Conclusions

This study found that filled prescriptions of MRA and loop diuretics were significantly less common in individuals with cognitive impairment. No difference regarding cognitive impairment was observed in terms of reaching target doses, but a higher age was associated with fewer filled prescriptions and less ability to reach the target doses of BBs. Our results suggest that individuals with cognitive impairment are partly undertreated for heart failure. The relatively low proportion of individuals reaching target doses is an important observation and indicates that the treatment of heart failure could be further optimised among older individuals.

### Electronic supplementary material

Below is the link to the electronic supplementary material.

Supplementary Material 1

## Data Availability

The datasets used and/or analysed during the current study are not publicly available since it contains personal sensitive data, in accordance to the EU’s data protection regulation, General Data Protection Regulation (GDPR), but are available from the corresponding author on reasonable request.

## Abbreviations

ACEI: angiotensin converting enzyme inhibitor
ADL: activity of daily living
ARB: angiotensin receptor blocker
ARNI: angiotensin receptor-neprilysin inhibitor
ATC classification: Anatomical Therapeutic Chemical classification:BB:beta blocker
CI: confidence interval
EQ-5D: quality of life according to EuroQuol five dimension
ESC: European Society of Cardiology
HFmrEF: heart failure with mildly reduced ejection fraction
HFrEF: heart failure with reduced ejection fraction
MARS-5: Medication Adherence Report Scale
MRA: mineralocorticoid receptor antagonist
NCD: neurocognitive disorder
NYHA: New York Heart Association
OR: odds ratio
SWENIS: Swedish National Inventory of Care and Health in Residential Aged Care
