# Supplementary material for: Use of heart failure medications in older individuals and associations with cognitive impairment
Source: BMC Geriatr. 2023 Aug 29;23:524. doi: 10.1186/s12877-023-04223-3 (PMC10463854; doi:10.1186/s12877-023-04223-3)
Supplement: Supplementary file 1 — Supplementary Material 1 [file 12877_2023_4223_MOESM1_ESM.docx]

Use of heart failure medications in older individuals and associations with cognitive impairment

Linnea Abramsson^1^, Annica Backman², Hugo Lövheim^3^, David Edvardsson^4^, Maria Gustafsson^1^

^1^Department of Integrative Medical Biology, Umeå University, 901 87 Umeå, Sweden

²Department of Nursing, Umeå University, 901 87 Umeå, Sweden

^3^Department of Community Medicine and Rehabilitation, Geriatric Medicine, Umeå University, Umeå, Sweden

^3^Wallenberg Center for Molecular Medicine, Umeå, Sweden

^4^School of Nursing and Midwifery, La Trobe University, Vic., 3084, Australia

**Supplementary information**

**Corresponding Author:**

Maria Gustafsson, Department of Integrative Medical Biology, Umeå University, 901 87 Umeå, Sweden Phone: +46 90 785 35 62

Email: maria.gustafsson@umu.se

| Table S1. Target doses of analysed drugs | |
| --- | --- |
| Drug | Target dose (daily dose) |
| Enalapril | 20–40 mg* |
| Ramipril | 10 mg |
| Candesartan | 32 mg |
| Losartan | 150 mg |
| Sacubitril/valsartan | 194 mg/206 mg |
| Bisoprolol | 10 mg |
| Carvedilol | 50 mg |
| Metoprolol | 200 mg |
| Spironolactone | 25 mg |
| Eplerenone | 25–50 mg* |

*The lowest doses are used as target doses in this study.
